# The E3 Ubiquitin Ligase Peli1 Deficiency Promotes Atherosclerosis Progression

**DOI:** 10.3390/cells11132014

**Published:** 2022-06-23

**Authors:** Fabienne Burger, Daniela Baptista, Aline Roth, Karim J. Brandt, Kapka Miteva

**Affiliations:** Division of Cardiology, Foundation for Medical Research, Department of Medicine Specialized Medicine, Faculty of Medicine, University of Geneva, Av. de la Roseraie 64, CH-1211 Geneva, Switzerland; fabienne.burger@unige.ch (F.B.); daniela.baptista@unige.ch (D.B.); aline.roth@unige.ch (A.R.); karim.brandt@unige.ch (K.J.B.)

**Keywords:** atherosclerosis, foam cells, VSMCs, plaque stability, ubiquitin ligase

## Abstract

Background: Atherosclerosis is a chronic inflammatory vascular disease and the main cause of death and morbidity. Emerging evidence suggests that ubiquitination plays an important role in the pathogenesis of atherosclerosis including control of vascular inflammation, vascular smooth muscle cell (VSMC) function and atherosclerotic plaque stability. Peli1 a type of E3 ubiquitin ligase has emerged as a critical regulator of innate and adaptive immunity, however, its role in atherosclerosis remains to be elucidated. Methods: Apoe^−/−^ mice and Peli1-deficient Apoe^−/−^ Peli1^−/−^ mice were subject to high cholesterol diet. Post sacrifice, serum was collected, and atherosclerotic plaque size and parameters of atherosclerotic plaque stability were evaluated. Immunoprofiling and foam cell quantification were performed. Results: Peli1 deficiency does not affect atherosclerosis lesion burden and cholesterol levels, but promotes VSMCs foam cells formation, necrotic core expansion, collagen, and fibrous cap reduction. Apoe^−/−^ Peli1^−/−^ mice exhibit a storm of inflammatory cytokines, expansion of Th1, Th1, Th17, and Tfh cells, a decrease in regulatory T and B cells and induction of pro-atherogenic serum level of IgG2a and IgE. Conclusions: In the present study, we uncover a crucial role for Peli1 in atherosclerosis as an important regulator of inflammation and VSMCs phenotypic modulation and subsequently atherosclerotic plaque destabilization.

## 1. Introduction

Atherosclerosis is a chronic immunometabolic vascular disease and a main cause of death in both developed and developing countries [1,2]. The fibrous cap is an atheroprotective layer of vascular smooth muscle cells (VSMCs) that covers the atherosclerotic plaque [3] and which rupture induces acute thrombo-occlusive events, such as myocardial infarction and stroke [4]. Immune cells and inflammation play a key role in promoting the disruption of the fibrous cap [3]. Amongst the many pathophysiological factors, are also abnormal cholesterol metabolism, endothelial dysfunction, and VSMCs phenotypic modulation, and systemic inflammation all contributing to the progression of atherosclerosis however some of the precise molecular mechanisms underlying the development of atherosclerosis are not fully understood. Ubiquitination is a multiple-step process of post-translational protein modification involved in the regulation of many important cellular processes. Emerging evidence suggests that ubiquitination could play important roles in the pathogenesis of atherosclerosis particularly interfering with the regulation of vascular inflammation, and endothelial and VSMCs cell function which could altogether have a major impact on atherosclerotic plaque stability [5]. In the present study, we investigate the role of Peli1, a member of the Peli (Pellino) family of E3 ubiquitin ligases in atherosclerosis pathology.

Peli1 has been found to be involved in the regulation of the toll-like receptor (TLR) and interleukin-1 receptor (IL-1R) signaling in innate immune cells [6,7,8]. In addition, Peli1 has been shown to modulate T cell receptor (TCR) signaling in T cells [8] and via control of TLR-mediated TRAF3 degradation and MAPK activation to trigger microglial activation and autoimmune inflammatory response [9]. Peli1 was also shown to be a critical regulator of the T cells activation as well as maintenance of peripheral T-cell tolerance [10]. Interestingly, Peli1 has been found to have a role in B cell autoantibody production in systemic lupus erythematosus pathogenesis, where via serving as an E3 ubiquitin ligase of NIK, it controlled Lys48-linked ubiquitination of NIK and subsequently noncanonical NF-κB activation [11]. Interestingly a recent publication has shown that Peli1 deficiency impairs the induction of the interleukin-1β (IL-1β) secretion by different NLRP3 triggers and it is required for NLRP3-induced caspase-1 activation and IL-1β maturation [12]. Peli1 conjugation to K63 ubiquitin chain to lysine 55 of the inflammasome adaptor apoptosis-associated speck-like protein containing (ASC), which in turn facilitates ASC/NLRP3 interaction and ASC oligomerization and subsequent inflammasome activation [12]. Since all the above-mentioned inflammatory signaling pathways particularly TLR, NF-κB, and NLRP3 activation have been shown by us and others to be major drivers of atherosclerosis disease progression and atherosclerosis pathology [13,14,15,16] on one hand and considering the involvement of Peli1 in their regulation, we investigated how Peli1 deficiency in an advanced model of atherosclerosis affects atherosclerosis disease progression.

Interestingly, the present study revealed that Peli1 plays a role of a critical regulator of systemic and local vascular inflammation, VSMCs foam cells formation which altogether impacts atherosclerotic plaque stability.

## 2. Materials and Methods

### 2.1. Mice

All mice were on a C57BL/6 background. Apoe^−/−^ mice were first crossed with Peli1^−/−^ mice and the resulting F1 mice were then backcrossed on the Apoe^−/−^ background. Eleven-week-old male Apoe^−/−^ C57Bl/6 and Apoe^−/−^ Peli1^−/−^ mice (crossed for 5 generations) were fed a high cholesterol diet (HCD) for 11 weeks (20.1% fat, 1.25% cholesterol, Research Diets, Inc., New Brunswick, NJ, USA) [17,18]. At sacrifice after overnight fasting, peripheral blood was collected by cardiac puncture. After the whole blood was clotted, it was centrifuged, and the serum was collected and stored at −80 °C until used. The systemic levels of cholesterol and triglyceride, nonessential fatty acids (NEFA), HDL (high-density lipoprotein), and glucose were evaluated using the in vitro tests for the quantitative determination LDL-Cholesterol Gen.3, HDL-Cholesterol Gen.4, TRIGL, and GLUC2, respectively (Roche Diagnostics GmbH, Mannheim, Germany). The measurement was performed using Cobas c 111 analyzers (Roche Diagnostics GmbH, Mannheim, Germany) at the University of Geneva Small Animal Phenotyping Core Facility. All experimental protocols and procedures were approved and performed according to the animal experimentation license (GE/100/16) issued by the Institutional Animal Care and Use Committee of the Geneva University School of Medicine. All procedures comply with the guidelines of Directive 2010/63/EU of the European Parliament on the protection of animals used for scientific purposes and the NIH Guide for the Care and Use of Laboratory Animals.

### 2.2. Flow Cytometry

Initially, the mice were anaesthetized with 4% isoflurane (induction) followed by 2% isoflurane (maintenance) for peritoneal fluid collection. After checking the effectiveness of the anaesthesia, 5 mL of 2% BSA PBS was injected with a 10 mL syringe and a 20G needle into the peritoneal cavity of the mice. After a short massage of the abdomen, about 4 mL of liquid containing the peritoneal cells was collected with the same syringe. The procedure was performed a second time under the same anaesthesia to obtain enough liquid peritoneal (LP) mononuclear cells. The syringes and needles were changed between each mouse. Spleens (SP) and lymph nodes (LN) (inguinal, mesenterics, brachial, axillary, and superficial cervical) were smashed mechanically, and the obtained cell suspension was passed through a 70 μm cell strainer (BD Biosciences, MD, USA). Erythrocytes were lysed and nucleated cells were washed twice and counted. Lymphocytes were plated in U-bottom 96-well plate (1 × 10^6^ cells/well) and stimulated in vitro with PMA (50 ng/mL; Sigma-Aldrich, St. Louis, MO, USA) and ionomycin (Sigma-Aldrich, St. Louis, MO, USA), in the presence of brefeldin A (Sigma-Aldrich, St. Louis, MO, USA) for 4 h. Before multi-color flow cytometry analysis to block nonspecific staining SP, LN or LP mononuclear cells (1 × 10^6^ cells per sample) were incubated with anti-mouse FcRIIB/FcRIIIA mAb (BD Bioscience, Franklin Lakes, NJ, USA) and then stained with fluorophore-conjugated antibodies—CD4 AF488 (1:100), clone RM 4–5; CD25 BV605 (1:55), clone M-A251; B220 AF488 (1:100), clone RA-3-6B2; IgM PE/CF594 (1:66), clone R6–60.2; CD43 APC (1:100), clone S7; CD5 PerCP (1:50), clone 53–7.3; CD1d PE (1:100), clone 1B1, (BD Bioscience, Franklin Lakes, NJ, USA), CXCL5 BV421 (1:100), clone 2G8 (BD Bioscience, Franklin Lakes, NJ, USA), PD-1 PE/CY7 (1:100), clone 29F.1A12, (BioLegend, San Diego, CA, USA), Bcl6 PerCP/CY5.5 (1:50), clone 7D1, (BioLegend, San Diego, CA, USA), CD23 BV421 (1:100), clone B3B4 (BioLegend, San Diego, CA, USA), CD21 PeCy7 (1:200), clone 8D9, (BioLegend, San Diego, CA, USA). For intracellular staining, the cells were fixed and permeabilized using the Cytofix/Cytoperm Plus Fixation/Permeabilization Kit (BD Biosciences, Franklin Lakes, NJ, USA) and stained with antibodies against FoxP3 PE CF594 (1:50), clone MF23, (BD Bioscience, Franklin Lakes, NJ, USA), and IL-17 RA APC (CD217) (1:100), clone PAJ-17R, (eBioscience, San Diego, CA, USA), T-bet BV605 (1:30), clone 4B10 (Biolegend, San Diego, CA, USA), GATA3 BV421 (1:30), clone L50–823, (BD Bioscience, Franklin Lakes, NJ, USA) and IL-4 (1:50) AF488, clone 11B11 (BD Bioscience, Franklin Lakes, NJ, USA). Using gates based on the forward and side scatter debris and dead cells were removed. After intracardial perfusion, the aorta was surgically excised and digested for 1 h at 37 °C in with Collagenase P, dispase, and DnaseI. The cell suspension was passed through a 40 μm cell strained and stained with LIVE/DEAD Fixable Near-IR Dead Cell Dye (1:500), Hoechst (1:200), and anti-mouse CD45 PE (1:100) (BD Bioscience, Franklin Lakes, NJ, USA). Flow cytometry analysis was used to evaluate VSMC transdifferentiation using anti-mouse CD68 PerCP/Cy5.5 (1:100) (Biolegend, San Diego, CA, USA), anti-mouse MAC2 PE/Cy7 (1:100) (Biolegend, San Diego, CA, USA), α-SMA Alexa Fluor 488 (1:50), clone 1A4 (Thermo Fischer, Waltham, MA, USA), Myh11 (1:50) (Thermo Fischer, Waltham, MA, USA) after excluding dead cells via LIVE/DEAD Fixable Near-IR Dead Cell Dye staining (1:500) (Thermo Fischer, Waltham, MA, USA) [19]. Samples were acquired in Gallios flow cytometer and analyzed using FlowJo software (BD, Version 10.0.8r1, Franklin Lakes, New Jersey, USA).

### 2.3. Immunohistochemistry

Mouse aortic sinuses were serially cut at 5 μm as previously described [20,21]. The obtained sections were fixed in acetone and immunostained with specific anti-mouse MMP-9 antibody (R&D Systems, Minneapolis, MN, USA), anti-mouse CD68 (Serotec, Puchheim, Germany). All sections were counter-stained with Mayer’s hemalum solution and rinsed in distilled water. Quantification was performed using the MetaMorph^®^ Microscopy Image Analysis Software from Molecular Devices or Definiens Developer 2.7 software (Definiens Inc., Carlsbad, CA, USA) for biomarker and morphological tissue profiling. The results were expressed as a stained area in lesion versus total lesion area.

### 2.4. Oil Red O Staining for Lipid Content

Five sections per mouse aortic roots and abdominal aorta were stained with Oil Red O, as previously described [20,21]. Aortas sections were counter-stained with Mayer’s hemalum solution and rinsed in distilled water. Quantifications were performed using the Definiens Developer 2.7 software (Definiens Inc., Carlsbad, CA, USA). Data were calculated as ratios of Oil Red Oil positive area staining (lipid content) versus total lesion area.

### 2.5. Sirius Red Staining for Collagen Content

Five sections per mouse aortic roots were stained with 0.1% Sirius red solution (Sigma-Aldrich, St. Louis, MO, USA) in saturated picric acid for 90 min at room temperature. Sections were washed with 0.01 M HCl for 1 min followed by water. After dehydration with ethanol for 30 s and cover-slipping, pictures of the sections were taken with ordinary polychromatic microscopy with identical exposure settings as previously described [22,23]. Quantifications were performed with Definiens Developer 2.7 software (Definiens Inc.). Data were calculated as ratios of Sirius red positive stained positive area staining versus total lesion area.

### 2.6. Multiplex Assay

Systemic cytokines and chemokines levels were measured with ProcartaPlex Mouse Cytokine/Chemokine Plane 1A plex (Thermo Fisher Scientific, Waltham, MA, USA), Cat. No. EPX360-26092-901) and ProcartaPlex Mouse Antibody Isotyping Panel (Thermo Fischer, Waltham, MA, USA) Cat. No. EPX070-20815-901 kits. The assays were performed according to the manual instructions and measured with calibrated Luminex Instrument (Luminex Corporation). xPONENT^®^ 4.2 for MAGPIX^®^ software (Luminex Corporation, Austin, TX, USA) was used for absolute quantification.

### 2.7. Statistical Analysis

For comparison of two groups of continuous variables, two-tailed unpaired Mann-Whitney U-tests with a confidence level of 95% were conducted if data were non-normally distributed using GraphPad Prism 9. The number of mice used for each analysis is indicated in the figure legends. All data are presented as median with interquartile range with *p* ≤ 0.05 *, *p* ≤ 0.01 ** and *p* ≤ 0.001 ***.

## 3. Results

### 3.1. Subsection

#### 3.1.1. Peli1 Deficiency Does Not Affect Cholesterols Levels, Atherosclerosis Lesion Burden, and Lipids Deposition in Apoe^−/−^ Mice on HCD

**Figure 1:**
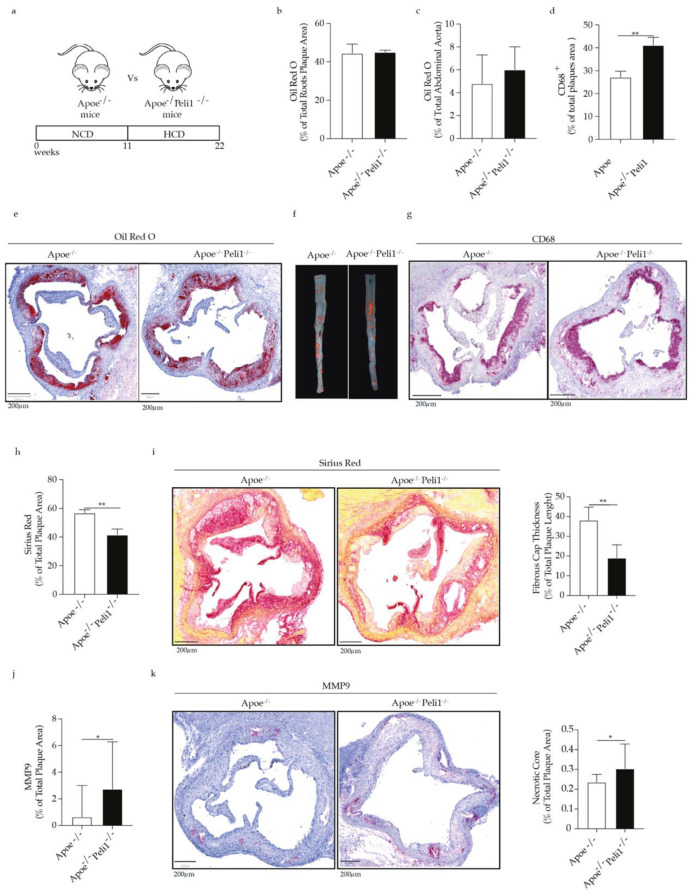
(**a**) Scheme illustrating the animal experimental settings. Bar graphs represent lipids accumulation as quantified by Oil Red O staining (**b**) in the aortic roots (**c**) in the abdominal aorta of Apoe^−/−^ and Apoe^−/−^ Peli1^−/−^ mice. (**d**) Bar graphs represent quantification of total CD68 macrophages present in the aortic roots of Apoe^−/−^ and Apoe^−/−^ Peli1^−/−^ mice on HCD. Representative images of (**e**) Oil Red O staining in the aortic roots, (**f**) Oil Red O staining in the abdominal aorta and (**g**) CD68 immunohistochemistry staining in the aortic roots of Apoe^−/−^ and Apoe^−/−^ Peli1^−/−^ mice. (**h**) Bar graphs and representative images showing quantification of collagen accumulation in the aortic roots of Apoe^−/−^ and Apoe^−/−^ Peli1^−/−^ mice fed HCD. (**i**) Bar graphs represent the quantification of the fibrous cap thickness in Apoe^−/−^ and Apoe^−/−^ Peli1^/−^ mice fed HCD. (**j**) Bar graphs and representative images showing the quantification of MMP9 expression in the aortic roots of Apoe^−/−^ and Apoe^−/−^ Peli1^−/−^ mice fed HCD. (**k**) Bar graphs represent the quantification of necrotic core area in the aortic roots of Apoe^−/−^ and Apoe^−/−^ Peli1^−/−^ mice, fed HCD, *n* = 7–8/group, U-Mann Whiney, all data are presented as median with interquartile range with *p* ≤ 0.05 * and *p* ≤ 0.01 **.

Apoe^−/−^ mice or Apoe^−/−^ Peli1 ^−/−^ mice were fed HCD for 11 weeks (Figure 1a). Peli1 deficiency in Apoe^−/−^ mice did not affect the levels of total cholesterol, LDL-C, triglycerides, NEFA, and HDL in Apoe^−/−^ mice (Supplementary Figure S1a–e). However, in comparison to Apoe^−/−^ mice on HCD, Peli1-deficient Apoe^−/−^ mice on HCD exhibited a significant reduction in glucose levels (Supplementary Figure S1e). Apoe^−/−^ and Apoe^−/−^ Peli1^−/−^ mice had a comparable increase in body weight after HCD feeding (Supplementary Figure S1g). Peli1-deficient Apoe^−/−^ mice fed HCD showed also a comparable with Apoe^−/−^ mice atherosclerosis lesion size and lipid accumulation in the aortic roots and the abdominal aorta (Figure 1b–f). However, the percentage of aortic roots CD68 macrophages was significantly higher in Peli1-deficient Apoe^−/−^ mice on HCD as quantified (Figure 1d) and evident in the representative picture of CD68 staining in the aortic roots (Figure 1g). Since high macrophages accumulation has been linked to plaque destabilization [24], we investigated the effect of Peli1-deficiency on markers of atherosclerotic plaque stability. Collagen plays a key role in atherosclerotic plaques stabilization and protection against rupture [25] and in comparison, to Apoe^−/−^ mice, Apoe^−/−^ Peli1 ^−/−^ mice fed HCD exhibited a significant reduction of collagen accumulation as quantified by Sirius red staining of aortic roots atherosclerotic lesions (Figure 1h,i). Importantly, in line with the reduction in the collagen content in Apoe^−/−^ Peli1^−/−^ mice on HCD, MMP9 expression was also elevated in the atherosclerotic roots of Apoe^−/−^ Peli1^−/−^ mice compared with Apoe^−/−^ mice (Figure 1j,k). Furthermore, Apoe^−/−^ Peli1^−/−^ mice fed HCD showed thinner fibrous cap and larger necrotic cores (Figure 1h,i). The present findings indicate that although Peli1 deficiency in Apoe^−/−^ mice in advanced atherosclerosis does not affect atherosclerotic lesion size, it promotes modulation of key features of the plaque stability.

#### 3.1.2. Peli1 Deficiency Affects Immune Cell Infiltration and VSMCs Foam Cells Formation in Atherosclerosis

**Figure 2:**
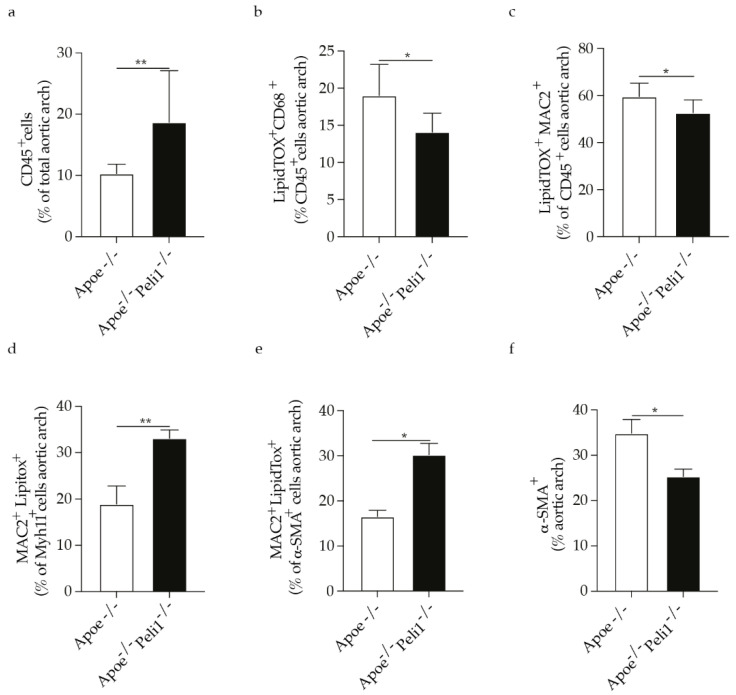
Bar graphs represent the quantification of aortic arch (**a**) total CD45^+^ cells in the aortic arch; (**b**) LipidTOX^+^ CD68^+^ cells as a percentage of CD45^+^ cells; (**c**) LipidTOX^+^ MAC2^+^ cells as a percentage of CD45^+^ cells; (**d**) MAC2^+^LipidTOX^+^ cells as a percentage of Myh11^+^ (VSMCs) cell; (**e**) MAC2^+^LipidTOX^+^ cells as a percentage of α-SMA^+^ (VSMCs) cells and (**f**) total α-SMA^+^ (VSMCs) cells in the aortic arch of Apoe^−/−^ and Apoe^−/−^ Peli1^−/−^ mice on HCD, with *n* = 7–8/group. U-Mann Whiney, all data are presented as median with interquartile range with *p* ≤ 0.05 * and *p* ≤ 0.01 **.

Since we observed a pronounced effect of Peli1 deficiency on atherosclerotic plaques stability we further investigated the extent on vascular inflammation and foam cell formation in the aortic arch of Apoe^−/−^Peli1^−/−^ mice fed HCD compared with Apoe^−/−^ mice. Apoe^−/−^Peli1^−/−^ mice fed HCD had a significant increase in the percentage of CD45 positive immune cells infiltrating the atherosclerotic arch and exacerbating the local vascular inflammation (Figure 2a). The percentage of CD68 or MAC2 foam cells (CD45 positive cells, hematopoietic origin) was quantified based on LipidTOX (marker of cellular lipid accumulation) positive staining. Interestingly, the percentage of CD68 or MAC2 foam cells was lower in Peli1 deficiency Apoe^−/−^ mice fed HCD (Figure 2b,c). VSMCs phenotypic switch and VSMCs foam cell formation are important factors relevant for atherosclerotic plaque stability and disease progression. Interestingly, the percentage of MAC2^+^ LipidTOX^+^ foam cells derived from VSMCs (CD45 negative cells expressing VSMCs contractile markers Myh11 and α-SMA) was pronouncedly increased in the aortic roots of Apoe^−/−^Peli1^−/−^ mice fed HCD in contrast to Apoe^−/−^ mice (Figure 2d,e). The Peli1-deficiency mediated effects on VSMCs foam cells formation were in line with the reduction in the percentage of α-SMA positive cells (Figure 2f) which altogether indicates that Peli1-deficiency has a major effect on VSMCs phenotypic modulation, which could further be linked to the observed atherosclerotic plaques vulnerability in Apoe^−/−^Peli1^−/−^ mice fed HCD compared with Apoe^−/−^ mice.

#### 3.1.3. Peli1 Deficiency Affects Immune Cell Response in Atherosclerosis

**Figure 3:**
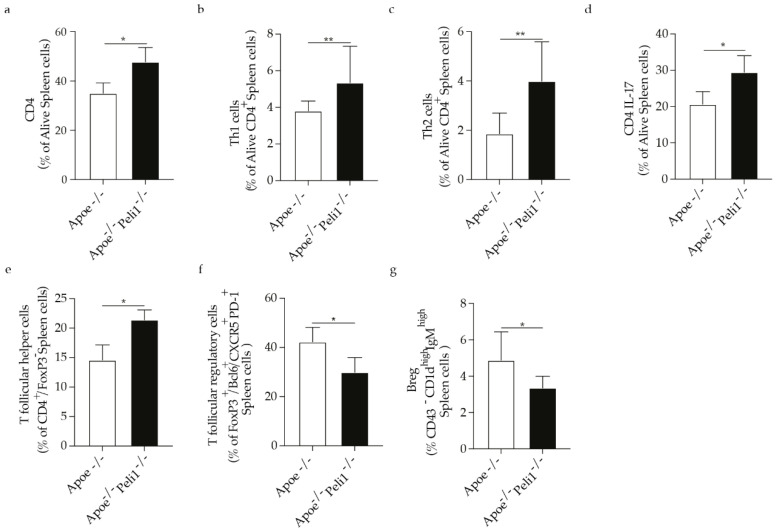
Bar graphs represent the quantification of the spleen (SP) (**a**) total CD4 cells; (**b**) Th1 cells; (**c**) Th2 cells; (**d**) CD4 IL-17 cells; (**e**) Follicular helper T cells; (**f**) Follicular regulatory T cells and (**g**) Breg cells in of Apoe^−/−^ and Apoe^−/−^ Peli1^−/−^ mice on HCD, *n* = 7–8/group, U-Mann Whiney, all data are presented as median with interquartile range with ± s.e.m with *p* ≤ 0.05 * and *p* ≤ 0.01 **.

**Figure 4:**
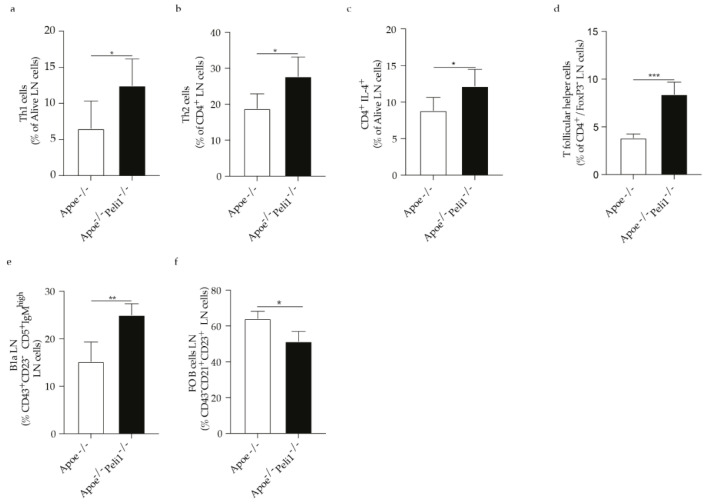
Bar graphs represent the quantification of lymph node (LN) (**a**) Th1 cells; (**b**) Th2 cells; (**c**) CD4 IL-4 cells; (**d** ) T follicular helper cells; (**e**) B1a cells and (**f**) FO B cells in Apoe^−/−^ and Apoe^−/−^ Peli1^−/−^ mice on HCD, *n* = 7–8/group. U-Mann Whiney, all data are presented as median with interquartile range with *p* ≤ 0.05 *; *p* ≤ 0.01 ** and *p* ≤ 0.001 ***.

**Figure 5:**
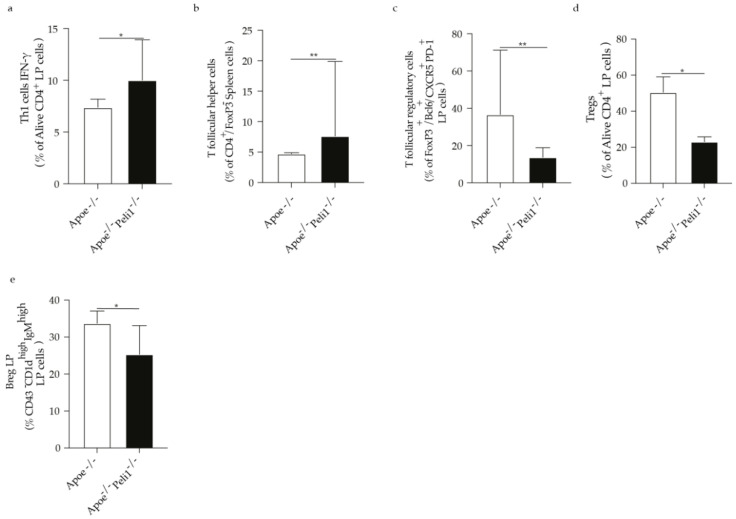
Bar graphs represent the quantification of liquid peritoneal (LP) (**a**) Th1 cells producing IFN-γ; (**b**) Follicular helper T cells; (**c**) Follicular regulatory T cells; (**d**) Tregs cells and (**e**) B reg cells in Apoe^−/−^ and Apoe^−/−^ Peli1^−/−^ mice on HCD, *n* = 7–8/group. U-Mann Whiney, all data are presented as median with interquartile range with *p* ≤ 0.05 * and *p* ≤ 0.01 **.

Considering that inflammation in atherosclerosis is largely controlled by the adaptive immune system and in line with the previous findings showing that specific effect of Peli1 on peripheral T-cells, we observed that Peli1-deficiency promoted an increase in CD4 cells, Th1, Th2, T cells secreting IL-17 as well as T follicular helper cells (Figure 3a–e). In parallel the percentage of follicular regulatory T cells and Breg cells in the spleen were significantly reduced in Apoe^−/−^ mice Peli1-deficient (Figure 3f,g). In line with the increase in the percentage of T cells in the spleen of Apoe^−/−^Peli1^−/−^ mice on HCD compared with Apoe^−/−^ mice on HCD exhibited a pronounced increase in the percentage of Th1, Th2, and CD4 cells producing IL-4 in the lymph node (Figure 4a–c) as well as induction of T follicular helper cells as well as B1a cells and follicular B cells were diminished (Figure 4e,f). In addition, Apoe^−/−^Peli1^−/−^ mice on HCD exhibited a pronounced increase in Th1 producing IFN-γ and T follicular helper cells present in the liquid peritoneal (Figure 5a,b), while the percentage of Treg cells and B reg cells was pronouncedly reduced in comparison to Apoe^−/−^ mice on HCD (Figure 5c–e). The presented data shows that Peli1 deficient in Apoe^−/−^ mice on HCD have a profound effect on systemic adaptive immune response promoting an increase in the pro-atherogenic immune cell populations like Th1 and T follicular helper cells and reduction in atheroprotective immune cells subsets like Treg cells and Breg cells.

#### 3.1.4. Peli1 Deficiency in Apoe^−/−^ Mice in Advanced Atherosclerosis Affects Systemic Inflammation

**Figure 6:**
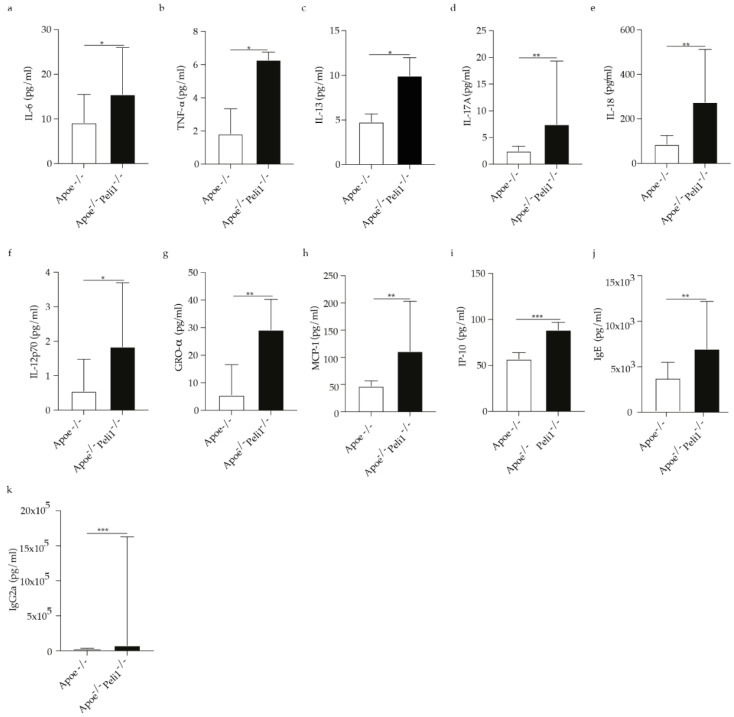
Bar graphs represent the quantification of systemic levels of (**a**) IL-6; (**b**) TNF-α; (**c**) IL-13; (**d**) IL-17; (**e**) IL-18; (**f**) IL12p70; (**g**) GRO-α; (**h**) MCP-1; (**i**) IP-10; (**j**) IgE and (**k**) IgG2a in Apoe^−/−^ and Apoe^−/−^ Peli1^−/−^ mice on HCD, *n* = 7–8/group, U-Mann Whiney, all data are presented as median with interquartile range with *p* ≤ 0.05 *; *p* ≤ 0.01 ** and *p* ≤ 0.001 ***.

Considering the importance of chronic inflammation in atherosclerosis, we investigated the impact of Peli1 deficiency on systemic immune mediators. The serum levels of cytokines and chemokines of Apoe^−/−^ and Apoe^−/−^Peli1^−/−^ mice fed HCD for 11 weeks were quantified. Apoe^−/−^Peli1^−/−^ mice on HCD exhibited an increase in the systemic level of IL-6 (Figure 6a) in comparison to Apoe^−/−^ mice on HCD. Furthermore, Peli1 deficient Apoe^−/−^ mice on HCD showed an increased level of TNF-α, IL-13, IL-17, and IL-18 as well as IL-12p70 (Figure 6b–f) in the serum versus Apoe^−/−^ mice. Furthermore, the systemic concentration of chemokines like GRO-α, MCP-1, and IP-10 (CXCL10) shown to contribute positively to the initiation and progression of atherosclerosis were increased systemically in Apoe^−/−^Peli1^−/−^ mice versus Apoe^−/−^ mice in advanced atherosclerosis (Figure 6g–i). These results suggest that Peli1 abrogation induces global systemic inflammation. Additionally, the immunoglobulin IgE is shown to contribute to atherosclerosis and obesity by affecting macrophages polarization and foam cell formation [26] was also elevated systemically in Apoe^−/−^Peli1^−/−^ mice versus Apoe^−/−^ mice in advanced atherosclerosis (Figure 6j). Peli1 has not only been linked to control of B cell autoantibody production, but Peli1-deficient mice showed elevated IgG2a [11], which is in line with our findings demonstrating that Peli1 deficient Apoe^−/−^ mice on HCD have significantly elevated IgG2a levels.

## 4. Discussion

The present study provides compelling evidence of the critical function of Peli1 E3 ubiquitin ligase in the pathogenesis of atherosclerosis. In advanced atherosclerosis, Peli1 deficiency appears to induce a multitude of detrimental effects. Although Peli1-abrogation did not affect atherosclerosis plaque size and cholesterol levels, Peli1 deficiency resulted in an elevation of the systemic inflammation including (1) induction of atherogenic immune cell populations—Th1 cells, CD4 L-17, and T follicular helper cells; (2) increased systemic pro-inflammatory cytokines like IL-6, TNF-a, IL-17, IL-18 and IL-12p70 as well as chemokines; (3) increased levels of circulating IgE and IgG2a. Moreover, Apoe^−/−^Peli-1^−/−^ mice exhibited several changes linked to atherosclerotic plaque destabilization like reduction of collagen accumulation and fibrous plaque thickness in parallel with an increased MMP9 expression and expansion of the necrotic core area. In parallel, Peli1 deficiency promoted VSMCs phenotypic modulation to macrophages like foam cells. Peli1 undoubtedly emerges as an important regulator of systemic and local vascular inflammation and which deficiency contributes to atherosclerosis progression linked to destabilization of the atherosclerotic plaques.

Inflammation has been proven to play a major role in all phases of atherosclerosis while ubiquitination could directly interfere with vascular inflammation, endothelial, and VSMCs cell function which could altogether affect important mechanisms of atherosclerosis disease development [5]. Peli1 has been found to be involved in the regulation of toll-like receptor (TLR) and interleukin-1 receptor (IL-1R) signaling in innate immune cells [6,7,8] and modulating T cell receptor (TCR) signaling in T cells [8] and trigger an autoimmune inflammatory response. [9] Peli1 has been shown to be a critical regulator of the T cells activation as well as maintenance of peripheral T-cell tolerance [10]. Moreover, deficiency in a ubiquitin ligase, Peli1, causes hyperactivation of T cells and rendered T cells refractory to suppression by T regulatory cells and transforming growth factor (TGF)-β [27]. In line with this finding, we demonstrated in the present study that Peli1 is a critical factor in the maintenance of peripheral T-cell tolerance since we observed a pronounced increase of pro-atherogenic immune cells populations like Th1 subsets [28] and the proatherogenic Th17 and IL-17 producing cells [29] as well as Th2 cells and T follicular helper cells in Apoe^−/−^Peli1^−/−^ mice fed HCD. The frequency of T follicular helper subsets correlates positively with laboratory parameters of atherosclerosis progression suggesting the essential role of T follicular helper cells in promoting inflammatory response and atherosclerosis progression [30]. The induction of T cells in secondary lymphoid organs would subsequently prompt T cells to migrate to the atherosclerotic lesions where they could be reactivated and be involved in the modulation of the immunoinflammatory response locally in the atherosclerotic plaques [31], however, T cells recirculation to atherosclerotic lesions has not been demonstrated in the present study. Furthermore, peritoneal inflammatory cells have been shown to play a pivotal role in the development of experimental atherosclerosis [32]. In this regard, we observed that Peli1 deficiency triggers pro-atherogenic Th1 cells producing IFN-γ [33,34] as well as follicular helper T cells [30]. The observed systemic increase in pro-atherogenic T cells upon Peli1 abrogation highlight the important role of Peli1 in immune tolerance. Moreover, Peli1 deficiency was linked to a reduction of atheroprotective regulatory T and B cell subsets [35,36]. Peli1 has been demonstrated to be involved in the control of B cell autoantibody production in systemic lupus erythematosus via a negative regulator of the noncanonical NF-κB pathway in B cells resulting in the production of more antibodies, specifically promoting the IgG2a class-switch in Peli1-deficient mice [11]. In the present model of atherosclerosis, hypercholesteremia in Peli1-deficient mice reduced the percentage of FO B cells in the lymph nodes of Peli1 deficient mice, while in line with the previous findings the systemic level of IgG2a has been significantly elevated in Apoe^−/−^Peli1^−/−^ mice fed HCD. Moreover, IgG has been shown to aggravate atherosclerosis [37]. In parallel, we observed increased systemic level of the immunoglobulin IgE in Apoe^−/−^Peli1^−/−^ mice shown to contribute to atherosclerosis and foam cell formation [26]. In line with this finding, we found that Peli1 deficiency exacerbates VSMCs phenotypic switch to foam cells. After lipid engulfment via scavenger receptors, VSMCs become foam cells [38,39] and acquire expression of macrophage markers like-CD68, F4/80, and MAC2 [40] which altogether could promote the destabilization of atherosclerotic plaques. It has been demonstrated that about 30–70% of the VSMCs with lost contractile phenotype express macrophages markers. Macrophage-like VSMCs exhibit inflammatory properties and perform phagocytosis and recruit circulating inflammatory cells exacerbating the plaque inflammation. VSMC-derived foam cells compared with macrophages foam cells show impaired phagocytic capacity and reverse cholesterol transport [41]. Macrophage-like VSMCs have also been shown to release MMPs and promote neutrophil recruitment and increase plaque vulnerability [40,42]. Indeed, we observed that increased VSMCs foam cells formation occurs in parallel with the induction of parameters of characterizing vulnerable plaques. It has been demonstrated that active inflammation is linked to thinning of the fibrous cap, predisposing the plaque to rupture [43]. In this regard, it appears that although Peli1-deficiency does not affect the atherosclerotic plaque size it plays an important role in the control of the systemic inflammation and therefore in plaque structure stability and the regulation of MMP-9, collagen and necrotic core expansion, and fibrous cap formation as parameters known to have critical clinical importance for atherosclerotic plaque rupture.

The association between inflammation and atherosclerosis has been actively investigated in the last three decades and the role of inflammation in the pathogenesis and progression of atherogenesis has been well established [44]. Endothelial dysfunction, oxidative stress, VSMCs macrophage foam cells accumulation, toll-like receptor signaling, NLPR-3 inflammasome formation, and subsequent pro-inflammatory cytokine production, such as TNF-α, IL-1β, IL-6 are few of the mechanisms implicated in the atherogenic process. Moreover, there is evidence that anti-inflammatory biologic drugs, such as anti-TNF-α and anti-IL1β agents, can decelerate the atherogenic process [45]. Peli1 has been found to be involved in the regulation of toll-like receptor (TLR) and interleukin-1 receptor (IL-1R) signaling in innate immune cells [6,7,8], while the present study showed that Peli1 deficiency in atherosclerosis promotes a storm of pro-inflammatory cytokines and chemokines which have been shown to not only direct leukocytes to the sites of inflammation during atherogenesis, but they also play a role in cell homeostasis and atherosclerosis plaque stability. Moreover, in line Peli1 abrogation triggers also an increase in CXCL10 which is strongly associated with a pronounced atheromatous formation [46].

The present study undoubtedly demonstrates that Peli1 abrogation in advanced atherosclerosis promotes atherosclerosis progression associated with induction of parameters characterizing destabilization of atherosclerotic plaques due to the lost control of the immune tolerance and increased in the systemic inflammatory cytokines and pro-atherogenic immunoglobulin.

## 5. Conclusions

Inflammation is crucial for atherosclerosis progression independently of hypercholesteremia and atherosclerotic plaque progression, but the mechanism and the factors regulating the inflammatory activation are incompletely understood. We demonstrate for the first time the role of the E3 ubiquitin ligase Peli1 as an important regulator for inflammatory-associated plaque destabilization in atherosclerosis.

## Data Availability

Not applicable.

## Supplementary Materials

The following supporting information can be downloaded at: www.mdpi.com/article/10.3390/cells11132014/s1, Figure S1: Bar graphs represent the quantification of systemic levels of (**a**) Total cholesterol, (**b**) LDL-C, (**c**) Triglycerides, (**d**) Non-esterified fatty acids (NEFA), (**e**) HDL, (**f**) Glucose and (**g**) Body weight on 11 of NCD or after 11 weeks of HCD in Apoe^−/−^ and Apoe^−/−^ Peli^−/−^ mice, n = 7–8/group, U-Mann Whiney, all data are presented as median with interquartile range with *p* ≤ 0.01 ** and *p* ≤ 0.001 ***.
