# Supplementary material for: The E3 Ubiquitin Ligase Peli1 Deficiency Promotes Atherosclerosis Progression
Source: Cells. 2022 Jun 23;11(13):2014. doi: 10.3390/cells11132014 (PMC9265341; doi:10.3390/cells11132014)
Supplement: Supplementary file 1 [file cells-11-02014-s001.zip › cells-1771739-supplementary.pdf]

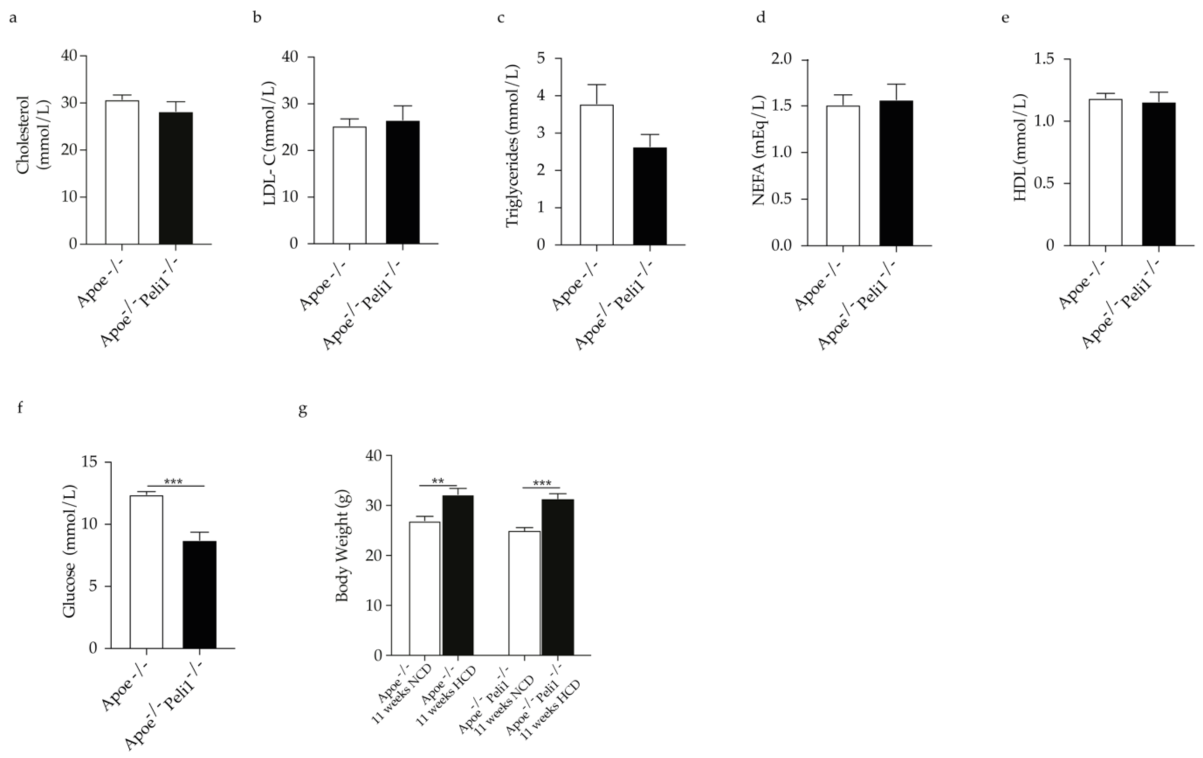

Supplementary Figure S1. Bar graphs represent the quantification of systemic levels of (**a**) Total cholesterol, (**b**) LDL-C, (**c**) Triglycerides, (**d**) Non-esterified fatty acids (NEFA), (**e**) HDL, (**f**) Glucose and (**g**) Body weight on 11 of NCD or after 11 weeks of HCD in Apoe$^{-/-}$ and Apoe$^{-/-}$ Peli$^{-/-}$ mice, n = 7–8/group, U-Mann Whiney, all data are presented as median with interquartile range with $p \leq 0.01$ ** and $p \leq 0.001$ ***.
